# Study protocol: incentives for increased access to comprehensive family planning for urban youth using a benefits card in Uganda. A quasi-experimental study

**DOI:** 10.1186/s12978-017-0400-8

**Published:** 2017-10-27

**Authors:** Afra Nuwasiima, Elly Nuwamanya, Patricia Navvuga, Janet U. Babigumira, Francis T. Asiimwe, Solomon J. Lubinga, Joseph B. Babigumira

**Affiliations:** 1 Global Health Economics Ltd, P.O Box 27011, Kampala, Uganda; 2 Pharmaceutical Outcomes Research and Policy Program, Department of Pharmacy, University of Washington, P.O. Box 357630, Seattle, WA 98195 USA; 3 Global Medicines Program, Department of Global Health, University of Washington, Seattle, WA 98195 USA

**Keywords:** Contraceptive use, Youth, Slums, Benefits card, Family planning, Corporate social responsibility, Impact evaluation, Economic evaluation

## Abstract

**Background:**

The use of contraception is one of the most cost-effective public health interventions and has the potential to prevent about 30% of maternal and 10% of child deaths in developing countries. Voucher-based initiatives for family planning are an effective and viable means of increasing contraceptive use. In this paper, we present a protocol for a pilot study of a novel incentive, a family planning benefits card (FPBC) program to increase uptake of family planning services among urban poor youth in Uganda while leveraging private sector funding.

**Methods:**

The study employs both impact and health economic evaluation methods to assess the effect of the FPBC program. We propose a quasi-experimental study design with two separate pre- and post-samples to measure program effectiveness. The main outcome of the impact evaluation is the percentage change in the prevalence of modern contraceptive use and unmet need for contraception. We will also conduct model-based incremental cost-effectiveness and budget impact analyses. The main outcomes of the economic evaluation are the cost per enrolled youth and cost per pregnancy averted, and cost per disability-adjusted life-year (DALY) averted. We will also pilot a corporate social responsibility model of sponsorship for the FPBC program in partnership with local corporations. Budget impact analysis will examine the potential affordability of scaling up the FPBC program and the fiscal implications of this scale up to the corporate social responsibility (CSR) budgets of partner corporations, the government, and the individual taxpayer.

**Discussion:**

In this study, we propose an impact and economic evaluation to establish the proof concept of using a FPBC program to increase uptake of family planning services among urban poor youth in Uganda. The results of this study will present stakeholders in Uganda and internationally with a potentially viable option for corporate-sponsored access to family planning in urban poor communities.

**Trial registration:**

MUREC1/7 No. 10/05-17. Registered 19th July 2017.

## Plain English Summary

The use of contraception contributes to the achievement of the Sustainable Development Goals (SDGs) by reducing the number of unplanned births and reducing maternal and child mortality. In low- income countries, the poor state of public health services and the multiple for-profit private providers mean that the cost remains a significant barrier to access to family planning services. The Government of Uganda is working towards achieving the family planning 2020 goals by closing down contraceptive use gaps but budget constraints remain a major impediment to these efforts. This paper presents an incentive-based family planning benefits card (FPBC) program to increase uptake of family planning services among urban poor youth in Uganda while leveraging private sector funding.

The protocol describes the health and economic evaluation processes that will be used to assess the impact of the FPBC program. The study will recruit youth of age 18-30 years and provide them with a family planning benefits card that will allow them access to modern family planning services in the intervention area. We will carry out household surveys among 670 women of age 18-30 years at 0 and 6 months respectively in both the intervention and control sites. The study will also conduct model-based cost-effectiveness and budget impact analyses. We will pilot test a corporate social responsibility sponsorship for the FPBC program.

If this study is successful, we will have provided stakeholders with a potential cost-effective model for increasing uptake of modern contraception in urban poor settings while leveraging on private sector funding.

## Background

The use of contraception is one of the most cost-effective public health interventions and has the potential to prevent about 30% of maternal and 10% of child deaths in developing countries [1]. In addition to limiting the adverse health effects of unintended pregnancies, contraception will contribute towards achieving the United Nations Sustainable Development Goals (SDGs) by reducing the number of unplanned births, reducing child morbidity and mortality, and increasing the resources that families and societies spend on the other necessities [2, 3]. Provision of universal access to modern contraceptives in Uganda would represent a highly cost-effective use of scarce healthcare resources [4]. According to the 2011 Uganda Demographic and Health Survey, the modern contraceptive prevalence rate was 26%, unmet need for family planning was 34%, and 43% of all pregnancies in the country were unplanned [5]. Women in the lowest wealth quintile had the highest levels of unmet need at 42% [5]. Additionally, previous studies have indicated that two of every five pregnancies in the country end in induced abortion [6] and notably, the higher number of unintended pregnancies in the country is due to lack of contraceptive use (88%) as compared to contraceptive failure (12%) [4].

In low-income countries like Uganda, the poor state of public health services and the multiple for profit-private providers mean that cost remains a significant barrier to access to family planning services especially to persons in the lowest wealth quintile who have the highest unmet need for contraception [7]. Other barriers to contraceptive access exist, including socio-cultural factors such as uncooperative spouses, perceptions of poor quality of services, and distance to services (especially in rural areas), all of which may disproportionally affect poor, young, and rural individuals [8–10]. Unmet need for family planning was higher among rural, less-educated and poor women than among well-educated, urban and wealthier women [11].

Studies have demonstrated the potential cost savings of modern contraception relative to the costs incurred by unintended pregnancies in Uganda. Reducing the unmet need for modern contraception would reduce maternal mortality by over 40 % and unplanned births and induced abortions by over 44 % while saving over three dollars for every dollar invested in reducing this unmet need [4, 12]. A cost-effectiveness analysis study reported that induced abortions that arose from unintended pregnancies were associated with $177 in societal costs, four-times higher than the level of per-capita health expenditure at the time [4, 13]. Fifty-two percent of the total societal costs were attributed to indirect costs and costs associated with productivity loss, while the remaining 48 % were associated with the direct costs of providing health care [4, 13].

The Government of Uganda acknowledges that limited access to family planning services hinders development and disenfranchises poor women and is committed to increasing contraceptive use by ensuring access to family planning services. To achieve this, the government has enlisted different strategies including the provision of integrated family planning services in all health facilities and procurement and distribution of contraceptives to men and women of reproductive age. However, budget constraints are a major impediment to these efforts [5]. Therefore, as the country works towards achieving the family planning 2020 goals by closing down contraceptive use gaps, strategies that increase the use of modern contraception and leverage the private sector are critical.

The concept of using benefits as a vehicle for health care access is not new in Uganda. Companies have, in recent years, provided medical benefits to their employees. Research suggests that provision of medical benefits helped companies retain good employees and improved on their performance and job satisfaction [14–17]. Incentives have been used to create demand for family planning services in the medical literature. In countries like Kenya, Pakistan, and Uganda, vouchers have been used to create demand for reproductive health services [18–20]. Earlier studies found voucher-based initiatives for family planning to be effective and recommended them as viable means of increasing contraceptive use in LMICs [20–24]. Tanzania is also currently implementing a free healthcare insurance benefits card for pregnant mothers [25].

In this paper, we present a protocol for a pilot study of a novel incentive benefits card system to increase uptake of family planning services leveraging the private sector. The proposed incentive system is called the Family Planning Benefits Card (FPBC) program. The FPBC program builds on the success of other voucher programs and is a partnership with hospitals and pharmacies. The FPCB program will target youth in the community using trained community health workers to provide counseling and guidance and recruit participants.

Unlike previous demand creation programs whose post-pilot financial sustainability was hinged on public-sector funding, the FPBC program proposes to use corporate social responsibility (CSR) as a form of funding for transition to scale. Corporations by their very nature have different incentives than governments: they are designed to maximize profit. However, many corporations include in their structure various levels of CSR. CSR is a form of corporate self-regulation integrated into the business model [26]. CSR goes beyond legal and regulatory compliance to further some social good, beyond the profit motive of the firm [27, 28]. CSR aims to encourage a positive impact on the environment and the community of consumers, employees, investors, and unrelated members of the general public. Corporations are incentivized to participate in CSR programs because they contribute to employee morale and performance [29], and may improve corporate profitability [30, 31]. In the setting of healthcare or public health, the aim of a corporation’s CSR activities might be to improve or to be seen to contribute to improving, health outcomes and public health in general.

A review of the literature suggests that activities related to CSR are rare in healthcare and public health in low-income countries. CSR has been suggested as a means to enhance the sustainability of immunization programs [32], prevent the environmental and health impact of the activates of oil companies [32, 33] and gold companies [34], increase access to water purification [35], and improve access to treatment of HIV/AIDS [36, 37]. We found no suggestion or application of CSR to enhance the sustainability of family planning programs in low-income countries, and Uganda in particular. Yet there is evidence that many local and multinational companies in Uganda have CSR budgets and engage in CSR activities, despite on-going challenges [38].

This paper describes the pilot implementation of a novel incentives-benefits card based model of increasing uptake of family planning in low-income settings. In performing this study, we hypothesize that the FPBC program will increase uptake of modern contraception and reduce the unmet need for contraception among the urban poor youth living in slums. Increased modern contraceptive coverage may lead to the reduction of maternal health-related deaths and consequently significantly reduce health care costs in the country. We also hypothesize that the FPBC program will be highly acceptable in the setting of urban slums. The study further hypothesizes that the FBPC program will be cost-effective from both the societal and payer (Uganda Ministry of Health) perspectives. If this program is cost-effective, we will have provided government and stakeholders with a potential cost-effective model for increasing uptake of modern contraception in urban poor settings. We also anticipate that corporate entities will be willing to commit funds that will pay for, and sustain the FPBC program.

## Methods

### Study objectives/aims

1. To evaluate the effectiveness of the FPBC program in increasing uptake of comprehensive family planning services among urban youth aged 18 to 30 years.
2. To evaluate the acceptability and usability of the FPBC program among the urban youth aged 18 to 30 years.
3. To estimate the cost-effectiveness and potential budget impact of the FPBC program on the taxpayer.
4. To pilot test a corporate social responsibility financing model that will pay for and potentially sustain the FPBC program.

### Intervention

This is a one-year pilot project implementation and evaluation study. We are proposing to design a novel incentive benefits card based system that will be used to increase uptake of family planning services. We will partner with a local insurance firm to design and manage the FPBC system. The research team will manage the evaluation of the effectiveness of the program and conduct other stakeholder engagements. In partnership with the local insurance firm and its partner clinics and pharmacies in the intervention area, the FPBC program will provide family planning services to the youth aged 18 to 30 years. We will recruit and train community health workers (CHWs) on the counseling and guidance necessary to recruit and access the family planning services. The CHWs will mobilize, sensitize, and recruit individuals in the community. Each recruited participant will be provided with a benefits card and a list of hospitals and pharmacies where the card can be used. The benefits card will contain the beneficiary’s photograph, names, and a unique identifier. The card will allow beneficiaries access family planning counseling and guidance, long-acting reversible contraception such as implants or intrauterine devices, hormonal contraception such as contraceptive pills, injections and vaginal rings and barrier methods such as condoms and diaphragms. The FPBC program will also provide free testing and care needed to inform the choice of family planning method. These tests will include among others pregnancy counseling and testing, referrals to primary health care providers for health conditions such as high blood pressure or diabetes, HIV counseling and testing, and screening and treatment of sexually transmitted infections. We will exclude permanent contraception, such as vasectomy and tubal ligation from our card benefits since our target group is expected, for the most part, to be interested in delaying as opposed to discontinuing fertility.

### Study setting

The study is being implemented in the city slums located in the suburbs of Kampala, Uganda’s capital city. The two city slums have been purposively identified for the pilot. The study sites are Kifumbira slum as an intervention area and Kalerwe slum as a comparator. Kifumbira slum is located northeast of Kampala city in Kampala Central division and neighbors Mulago to the west, Bukoto to the east, Kyebando to the north and Kamwokya to the south. Kalerwe slum is located north of Kampala city in Kawempe division. The two slums have populations that share the socio-demographic characteristics and hence provide a good intervention-comparison pair for the study. The majority of the two slum’s residents fall in the lowest wealth quintile and record the high levels of unmet need for contraception — 42% as compared to the national average of 34% [5]. Studies also suggest that only about 24% of the slum population is engaged in regular jobs, an indication that majority of the residents are unable to afford basic medical care [39].

### Study design

The proposed study is a *pilot* project implementation, and prospective impact and economic evaluation. The project includes three main complimentary sub-studies: 1) an impact evaluation, 2) an acceptability and usability study, and 3) model-based health economics evaluation.

### Impact evaluation study (objective 1)

#### Study design and participant recruitment

**Fig. 1:**
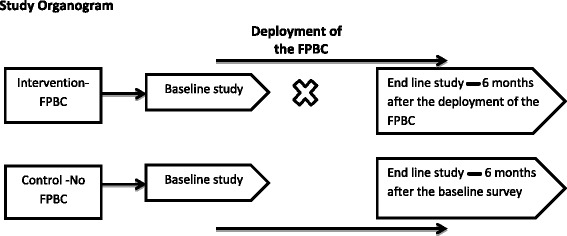
Study organogram. Quasi-experimental study design with a comparator, and a pre- and post –FPBC program separate sample surveys

The impact evaluation will utilize a pre- and post- quasi-experimental study design with a control group. We propose a household quantitative survey both in the intervention and control sites to assess the study outcomes at 1 and 6 months respectively. The impact evaluation study will have two primary outcomes: percentage change in the prevalence of modern contraceptive use and the percentage change in the prevalence of unmet need for contraception by study group. The secondary outcomes will include the percentage change in the knowledge of different methods of family planning, and satisfaction levels with the services provided among the study groups. The household survey shall only be conducted among females that are aged between 18 and 30 years, residents of the study areas, and willing to provide informed consent to participate in the survey (Fig. 1).

We performed a mapping exercise at the start of the project aimed at delineating the study area boundaries and generating a list of households with members meeting the study’s inclusion criteria. We also identified the clinics and pharmacies in the Kifumbira slum that provide family planning services. A two-stage sampling technique will be used to select the study samples. The first stage involves purposively selecting the two study sites for the evaluation that are similar by both population characteristics and availability of family planning providers. The next stage will be randomly selecting the households that shall work as primary sampling units. From the compiled household sampling frame, a computer-generated random sample will be chosen. Sampled households will then be approached in their homes and invited to participate in the study. In every sampled household, all eligible participants for the survey will be invited to participate in the study. The survey tool will be administered with the help research assistants in a private setting. The same sampling approach will be reciprocated at end-line study leading to two separate samples per study site.

#### Household survey sample size

According to the 2011 Uganda Demographic Health Survey (UDHS), the prevalence of modern contraception is 26% and the prevalence of unmet need for contraception among women in the lowest wealth quintile is 42%. The study’s aim is to cause an increase in the prevalence of modern contraception use by about 10 percentage points by the end of the program. The study utilizes the sample size formula for behavioral studies [40] stated below. The prevalence of modern contraception has been used to estimate the sample size as described below.

$$ n=d\left(\frac{{\left({Z}_{1-\alpha /2}\sqrt{2p\left(1-p\right)}+{Z}_{1-\beta}\sqrt{\left({p}_1\left(1-{p}_1\right)+{p}_2\left(1-{p}_2\right)\right)}\right)}^2}{{\left({p}_2-{p}_1\right)}^2}\right) $$

Where:

*n* =sample size.

*d* = design effect.

*p*_1_ = the prevalence of modern contraception.

*p*_2_ = projected prevalence at the end of the study, so that (*p*_2_ − *p*_1_) is the magnitude of change you want to be able to detect.

*p =* (*p*_1_ +*p*_2_)/2.

*Z*_1 − *α*_= the z-score corresponding to desired level of significance.

*Z*_1 − *β*_ = the z-score corresponding to the desired level of power.

The Table 1 below shows the estimated sample sizes for each group with varying percentage points of the magnitude of change to be detected, different power sizes, and two-sided level of significance of 0.05. A sample size of 669 participants per group will be used for the baseline and end-line surveys in both the control and the study groups, in order to obtain 80% power to detect a 10% change in the prevalence of modern contraception at a two-sided 5% level of significance. The study’s total sample size in both the groups will be 1338 participants.

**Table 1:** Sample size estimation

Power (1 − *β*)	Percentage change in proportions			
	5%	10%	15%	20%
0.80	2557	669	308	178
0.85	2924	765	352	204
0.90	3422	895	412	238
0.95	4231	1106	509	293

#### Household survey instrument

The survey tool will contain sections on socio-economic and demographic characteristics (such as age, education, household income and expenditure, employment, marital status, religion etc.), reproduction (gravidity, parity, age at first sex, number of under-five children etc.), access to family planning information (sources of information e.g. radio, television, health workers etc.), history of family planning, current use of modern contraception, desire to stop or delay pregnancy (unmet need), reasons for choosing family planning methods, perceived barriers to family planning access and satisfaction levels with provided services and the FPBC program. Questions on socio-economic and demographic characteristics, reproduction, current use and unmet need for modern contraception will be modified from the 2011 UDHS questionnaires.

#### Data collection and analysis for the household survey

We will carry out data collection for the house survey at both baseline and end-line using the personal digital assistant (PDA) data collection systems. We propose to use open data toolkit (ODK) for questionnaire design and data collection, and a dedicated server to receive data in real time. The electronic system will have data checks and necessary skips in order to improve data quality. The data from the server will be downloaded in excel format and sent to respective software for further analysis. The analysis will be done using R studio (open source version 1.0.136, R Foundation for Statistical Computing, Vienna, Austria) and STATA version 13.0 (Stata Corporation, College Station, Texas, USA).

#### Primary analysis for impact evaluation

To assess if the control group is as similar as possible to the intervention group in terms of pre-intervention characteristics, we will compare the observed demographic and socioeconomic characteristics by group. Group differences for each variable will be summarized using means and proportions. In the event that the observed baseline characteristics show no similarity, we will use propensity score matching to identify the region of common support and discard observations that are substantially dissimilar in the analysis. The primary analysis of this evaluation is an intention to treat analysis (ITA). The magnitude of change in proportion between the two groups provides a potentially unbiased estimate of the average treatment effect (ATE) of the FPBC program. We will use a two-sample proportion z-test to test the null hypothesis that the two sample proportions are significantly different at six months between the intervention and control groups at 5% level of significance. We will also carry out a difference in difference (DID) analysis to identify the magnitude of change as a result of the program if any.

#### Secondary analysis

We shall conduct a number of secondary analyses. We propose to assess the effect of the intervention on the participant’s change in family planning knowledge and satisfaction with the services using two-sample proportion z tests. Comparisons of proportions of changes in knowledge of different methods of family planning and satisfaction levels by intervention group will be conducted. Binary logistic regression analysis using modern contraception use as the outcome variable and demographic characteristics, satisfaction, and knowledge as explanatory variables will be conducted. Both adjusted and crude odds ratios with their corresponding confidence intervals and *p*-values will be reported. All results will be considered statistically significant at a 5% level of significance.

### Acceptability and usability of the FPBC program study (objective 2)

#### Study design, setting, and participant recruitment

Upon finishing the baseline survey, we will embark on the process of mobilizing study participants in the Kifumbira slum to enroll for the FPBC program. The acceptability study will utilize a mixed (quantitative and qualitative) methods study. The quantitative part will use data from the recruitment forms (acceptability forms) and the family planning medical records form (usability form), and the qualitative part will use data from the key informant interviews (KIIs) and Focus group discussions (FGDs).

#### Subject selection and sample size for the acceptability and usability study

This study will approach study participants from their homes and invite them to enroll into the FPBC program. Recruitment will be done by moving from house to house and screening youth for eligibility and willingness to participate into the program. This will be done with the help of CHWs and the study’s trained research assistants. All persons eligible for the study will first be taken through a counseling and guidance session about family planning before enrolling into the program. Only participants; 1) aged between 18 to 30 years, 2) self-reported non-use of contraception methods, 3) for females, not currently pregnant, and 4) willing to provide consent will be considered eligible to receive the FPBC will be selected to participate into the program. We propose to recruit a purposive sample of 300 participants for the family planning benefits card for this pilot. A total of 250 persons will be females and 50 will be males of the same age brackets. This sample is based on resource availability for the study. The inclusion of men will help the study team to test a way of attracting males to embrace contraceptives use.

Persons meeting the stated inclusion criteria and accepting to enroll for the FPBC program will be requested to provide passport photograph and their biodata. All recruited members shall then be provided with the FPBC that shall contain their full names and a photograph, and a list of clinics and pharmacies where the FPBC can be used. They will then be guided on how to use this card to visit the selected licensed hospitals and pharmacies to access free family planning services. The FPBC will have unique numbers that shall be used to track the use of the card at the clinics and pharmacies. An updated list of the beneficiaries shall also be provided to the service providers for cross-validation to avoid misuse of the card by the non-beneficiaries. Trained community health workers will continuously visit the enrolled participants and provide them with counseling and guidance.

#### Measurement of acceptability

Acceptability will be quantitatively measured by estimating the proportion of eligible participants who accept to enroll into the program. Persons who refuse to join the FPBC program will be probed further to identify reasons for refusal. Quantitative categories for reasons for refusal will be created for analysis purposes.

#### Measurement of usability

Usability of the FPBC will be measured by the number of times the card beneficiaries visited the service providers to seek care. The data will be disaggregated by the type of method and sex. This data will be generated from the health utilization reports generated from the medical records.

#### Data collection for acceptability and usability study

Data for the acceptability will be collected with the help of trained research assistants exactly after the baseline. Approached participants will be requested to complete a study recruitment form. This form shall also be completed by those declining to join the program to help the study understand reasons for declining to join the program. The study will also collect usability data using the family planning medical records form that shall be completed at the clinics and pharmacies. Focus group discussions and key informant interviews will also be carried to provide a detailed understanding of the program achievements and challenges. Trained qualitative research assistants shall be used to collect qualitative data.

#### FPBC study instruments

##### Recruitment form (acceptability form)

All enrolled participants will complete recruitment form. The recruitment form will capture participant’s demographics and an acceptability question. For those who will accept to enroll into the program, more personal details such as names, phone contacts, and passport photograph will be collected.

##### FPBC medical records form

In partnership with a local insurance firm, we propose to design the FPBC medical records form (FMRF) which will be given to the partner clinics and pharmacies to collect data on clients such as date, benefits card number, purpose of the visit, services received, new or continuing clients, nature of contraceptive supplies given, need for referral and any notes taken on the client. Each provider will be trained on the implementation of this routine medical data collection form and other project activities. These forms shall be completed in triplicate. A copy shall be retained by clinics and pharmacies, and other copies given to the local insurance firm provider and research team. This will make monitoring the card usage by the research team credible. Data from the FMRF will be used to assess the usability of the card services. We will at the end of the study match the usability and acceptability data sets for analysis of the acceptability and usability of the FPBC program.

##### Key informant interview

We propose to conduct 15 key informant interviews (KIIs) with the community health workers and health facility workers implementing the program. The KIIs will be conducted at the end of project implementation to identify the implementation challenges, and document successes realized during the study period. The respondents will also be asked to make recommendations to help the team make improvements on the program.

##### Focus group discussions

We also propose to conduct 6 focus group discussions (FGDs) of 8-10 participants per group in the intervention group at the end of the program. The FGDs will be disaggregated by sex, age, and acceptance of participation in the FPBC program. The FGDs will inform knowledge of youth in slum communities about family planning, their attitudes, practices, and barriers towards family planning, drivers of youth choices towards the use of certain family planning methods, health workers’ perceptions about the program, including its benefits, challenges experienced, and suggestions improvement of the program. Persons that declined to participate in the program will be probed further to identify reasons for refusal.

#### Data collection and analysis for objective 2

##### Quantitative analysis

All the data collected from the recruitment forms and FMRF will be coded and double entered in Epidata version 3.1. Consistency checks and cross-validation will be done at data entry to remove discrepancies in the final datasets. The analysis will be done using R studio (open source version 1.0.136, R Foundation for Statistical Computing, Vienna, Austria) and STATA version 13.0 (Stata Corporation, College Station, Texas, USA) .

We will perform descriptive analyses of the demographic characteristics of participants included in the study using means and proportions. Univariate and bivariate analyses will be performed to further characterize the study sample. We will report means and proportions of the different variables such as the mean number of times of the card usage per method, proportions of different contraception methods undergone and will be disaggregated by sex and age group. We will explore the association between different variables and acceptability of FPBC program, usability of the card services and the reasons for declining screening using the chi-square test. Binary logistic regression will also be conducted with acceptability (accepted vs. refused) as the outcome variable. We will also perform poison regression analysis with number of times card used as the outcome variable against the participant’s socio-economic demographic characteristics.

##### Qualitative analysis

Qualitative interviews will be audio recorded and notes will be taken during the interviews. The recordings will be transcribed verbatim and translated into English if the recording is in the local language. Analysis will be done using Atlas Ti software. Different themes will be identified and logged into the analytical frame work and will be used to develop code definitions, code categories and themes. Transcripts will be entered into Atlas Ti scientific software as primary documents and free codes generated based on the codebook while allowing for open coding where new codes emerge. Magnitudes of responses as well as patterns in the data using code frequencies in the codes-primary documents table will be identified. Key themes identified in the discussions will be reported.

### Model-based health economic evaluation study of the FPBC program (objective 3)

We propose to conduct a model-based incremental cost-effectiveness analysis (CEA) and budget impact analysis (BIA). We will develop a model of the cost-effectiveness of the FPBC program using Microsoft Excel. The main outcomes of the economic evaluation are the cost per enrolled youth, cost per pregnancy averted and cost per DALY averted. We also propose to perform a static aggregate budget impact analysis (BIA) to examine the potential economic value of scaling up the FPBC program and the fiscal implications of this scale up to the CSR budgets of partner corporations, to the government, and to the individual tax payer of implementing this FPBC program.

We will perform a primary micro-costing of FPBC program from the payer’s perspective i.e. include all direct medical and all direct non-medical costs and the modified societal perspective i.e. add indirect costs of lost productivity [41]. The direct medical costs will include all resources used in providing family planning services in terms of the costs of personnel, medical devices, and supplies. The direct non-medical costs will include transport and any upkeep visiting the family planning clinics and pharmacies and opportunity costs. Data on direct non-medical costs will be collected through follow-up studies. Data on personnel resource use will be collected from the benefits card claims records. All hospital and pharmacy visits will be timed to estimate participant time use as well as potential lost work time. Data on wages will be used to estimate indirect costs. A time-motion and costs survey will be carried out among program beneficiaries to estimate the direct non-medical costs.

The model of cost-effectiveness will be validated by varying inputs to check if the expected values are logical. We will also set costs and outcomes to 0 separately and check if identical expected values are obtained for the comparators. The baseline analysis will consist of the calculation of the costs, the proportion of participants on family planning by contraception method, and the incremental cost-effectiveness ratios—cost per youth enrolled, and cost per method. Sensitivity analyses will be performed to investigate the impact of parameter uncertainty on payoffs. Empirically derived confidence intervals will be used when available. One-way sensitivity analyses will be performed on all parameter inputs to generate tornado diagrams representing the most influential parameters. Distributions will be assigned to all input parameters and probabilistic sensitivity analysis will be performed using a second-order Monte Carlo simulation. This will enable the generation of a cost-effectiveness acceptability curve to represent the impact of parameter uncertainty on estimates of cost-effectiveness.

We will also estimate the aggregate national budget impact as well as the cost per Ugandan taxpayer per year of implementing a possible FPBC program. The BIA will involve the following steps: 1) characterization of the population of Uganda to estimate the potential number of people to be impacted by a possible FPBC program, 2) selection of a time horizon between 1 and 3 years, 3) documenting the current state of family planning nationally, 4) estimating the cost of FPBC program at scale, and 5) presenting budget impacts tailored to different audiences. The main analysis will be the generation of an estimate of the potential annual fiscal implication for the CSR budgets of potential corporate sponsors.

### Corporation-sponsored sustainability drive (objective 4)

The aim of the corporation-sponsored sustainability drive is to pilot test a way for funding a possible FPBC program beyond the immediate pilot program phase. Our vision is that corporate entities, through their CSR budgets, will be willing to fund an effective and cost-effective FPBC program. The first three aims of this study will provide effectiveness and economic efficiency data and the fourth aim will pilot test our idea of corporate partnership. The study will identify corporations with CSR budgets and approach them for potential partnership. The study’s media and publicity team will work with our corporate partners to design a media and community publicity plan to enable their good work to find an audience. We will design different metrics to reward and recognize different corporations depending on the expenditure on the FPBC program. All metrics for recognition will be adjusted based on corporations published book value or market capitalization to incentivize a wide range of small, medium, and large corporations.

### Study status

The study has so far recruited and trained 10 community health workers (CHWs) in the study areas to continuously sensitize the youth on family planning services and the associated benefits. Mapping of the study area to delineate boundaries and compile household lists has been completed. A total of six health facilities have also been enlisted for partnership to provide family planning services. The local medical insurance firm (benefits provider) has also been identified and partnerships discussions finalized.

## Discussion

This paper describes the protocol for a pilot project implementation, a quasi-experimental impact evaluation, and economic evaluation of a family planning benefits card program. The impact evaluation will help the research team establish the proof of concept of provision of family planning benefits to poor urban youth in Uganda. The results from the model-based health economic evaluation will provide estimates the economic value provided by the program. The potential budget impact analysis of the program will provide estimates of the affordability and potential fiscal impact to multiple stake holders of the program.

### Innovation and impact

The proposed study will assess the impact of a family planning benefits card on uptake of modern contraceptives targeting the urban poor with the highest unmet need for contraceptives and that are unable to afford these services. We hypothesize that this program will be highly acceptable and has the potential to reduce unintended pregnancies by over 50% in the study area over the one-year project period. Unlike other programs whose post-pilot financial sustainability is hinged on public sector funding, this study is proposing to use corporate social responsibility financing as a form of funding on the transition to scale. We anticipate that if the program is effective and efficient, corporate companies through their CSR budgets would be willing to support it.

If the proposed study is successful, we will have presented to the stakeholders in Uganda, and internationally with a potentially viable option for a corporate-sponsored benefits system to increase uptake of family planning in urban poor communities. The FBBC program might also expand the services coverage to include all sexual and reproductive health services and scale up the program to all the country’s urban slums, refugee camps, and fishing communities that record high levels of poverty and unmet need for contraception.

### Study strengths and limitations

Our study protocol employs rigorous evaluation methods to measure the effect of the FPBC program. The study proposes to use a quasi-experimental study design with a separate pre and post survey sample in each study group. This design will make it possible for the study to test causal hypotheses and detect any differences arising out of the program..

The study is limited to only youth aged 18 to 30 years living in urban slums. As a result, the study’s findings may not be generalizable to other groups of women, and in other study settings. Also, quasi-experimental designs by definition lack random assignment. This characteristic comes with challenges of confounding variables that pose threats to the study’s internal validity.

## Conclusion

In this paper, we describe planned impact and economic evaluation methods to assess a family planning benefits program to increase uptake of family planning services among the urban youth aged 18 to 30 years in poor urban areas in Uganda. The study will establish the proof concept of using the benefits program to increase uptake of family planning services. The study will also asses the efficiency and potential affordability of the program to different stake holders. The final part of the study will assess the feasibility of using corporate social responsibility financing as a form of transition to scale of the program.

## Abbreviations

ATE: Average treatment effect
BIA: Budget impact analysis
CEA: Cost effectiveness analysis
CHWs: Community health workers
CSR: Corporate Social Responsibility
FGDs: Focus group discussions
FMRF: FPBC Medical Records Form
FPBC: Family planning benefits card
IRB: Institutional review board
ITA: Intention to treat analysis
KIIs: Key informant interviews
MUST: Mbarara University of Science and Technology
ODK: Open data toolkit
PDA: Personal digital assistant
SDGs: Sustainable development goals
UDHS: Uganda Demographic and Health Survey

## References

[1] Azmat SK, Ali M, Ishaque M, Mustafa G, Hameed W, Khan OF, Abbas G, Temmerman M, Munroe E (2015). Assessing predictors of contraceptive use and demand for family planning services in underserved areas of Punjab province in Pakistan: results of a cross-sectional baseline survey. Reprod Health.

[2] Okonofua F (2006). Abortion and maternal mortality in the developing world. J Obstet Gynaecol Can.

[3] Uganda Bureau of Statistics (UBOS) and Macro International Inc (2007). Uganda demographic and health survey 2006.

[4] Babigumira JB, Stergachis A, Veenstra DL, Gardner JS, Ngonzi J, Mukasa-Kivunike P, Garrison LP (2012). Potential cost-effectiveness of universal access to modern contraceptives in Uganda. PLoS One.

[5] Uganda Bureau of Statistics (UBOS) and ICF International Inc (2012). Uganda demographic and health survey 2011.

[6] Mirembe FM (1996). A situation analysis of induced abortions in Uganda. African Journal of fertility sexuality and reproductive health.

[7] Singh S, Darroch JE, Ashford LS. Adding it up: The costs and benefits of investing in sexual and reproductive health 2014. Guttmacher Institute, New York, 2014. Available from: https://www.guttmacher.org/sites/default/files/report_pdf/addingitup2014.pdf.

[8] Stephenson R, Hennink M (2004). Barriers to family planning service use among the urban poor in Pakistan. Asia-Pac Popul J.

[9] Eva G, Quinn A, Ngo TD. Vouchers for family planning and sexual and reproductive health services: a review of voucher programs involving Marie Stopes international among 11 Asian and African countries.Int J Gynecol Obstet. 2015;130(S3). Available from: http://onlinelibrary.wiley.com/doi/10.1016/j.ijgo.2015.06.023/full 10.1016/j.ijgo.2015.06.023 26165906

[10] Ouma S, Turyasima M, Acca H, Nabbale F, Obita KO, Rama M, Adong CC, Openy A, Beatrice MO, Odongo-Aginya EI, Awor S (2015). Obstacles to family planning use among rural women in Atiak health center IV, Amuru District, northern Uganda. East Afr Med J.

[11] Guttmacher Institute. Contraception and Unintended Pregnancy in Uganda: Fact Sheet. 2013. Available from: https://www.guttmacher.org/sites/default/files/factsheet/fb-contraception-and-unintended-pregnancy-in-uganda.pdf

[12] Vlassoff M, Sundaram A, Bankole A, Remez L, Mugisha F. Benefits of meeting the contraceptive needs of Ugandan women. Issues Brief (Alan Guttmacher Institute). 2009;(4):1–8. 19938236

[13] Karra M, Gribble JN (1996). Costs of induced abortion and cost-effectiveness of universal access to modern contraceptives in Uganda. Health.

[14] O'brien E (2003). Employers' benefits from workers' health insurance. The Milbank Quarterly.

[15] Hameed A, Ramzan M, Zubair HM. Impact of compensation on employee performance (empirical evidence from banking sector of Pakistan). Int J Bus Soc Sci. 2014;5(2).

[16] Yousaf S, Latif M, Aslam S, Saddiqui A (2014). Impact of financial and non-financial rewards on employee motivation. Middle-East J Sci Res.

[17] Nzyoka CM, Orwa BH (2016). The relationship between Total compensation and employee performance in the insurance industry, case of Mayfair insurance company limited. Psychology and Behavioral Sciences.

[18] Arur A, Gitonga N, O’Hanlon B, Kundu F, Senkaali M, Ssemujju R. Insights from innovations: lessons from designing and implementing family planning/reproductive health voucher programs in Kenya and Uganda. Bethesda: Private Sector Partnerships-One project, Abt Associates Inc. 2009 Nov:23-38. Available from: https://academic.oup.com/heapol/article/26/1/25/626276.

[19] Nuccio BO, Reichwein B. Understanding clients and achieving FP2020 goals : exit interviews help deliver client-centred services. Marie Stopes International; Research Brief Series. Available from: https://mariestopes.org/media/2125/understanding-clients-and-achieving-fp2020-goals.pdf

[20] Menotti EP, Farrell M (2016). Vouchers: a hot ticket for reaching the poor and other special groups with voluntary family planning services. Global Health: Science and Practice.

[21] Bellows B, Bulaya C, Inambwae S, Lissner CL, Ali M, Bajracharya A (2016). Family planning vouchers in low and middle income countries: a systematic review. Stud Fam Plan.

[22] Bellows B, Bajracharya A, Bulaya C, Inambwae S. Family planning vouchers to improve delivery and uptake of contraception in low and middle income countries: a systematic review. Available from: http://www.popcouncil.org/uploads/pdfs/2015RH_FP-VouchersReview.pdf 10.1111/sifp.12006 PMC5434952 27859338

[23] Eva BG, Shah S, Quinn A, Ngo T. Are our voucher programmes working ? Evaluating our methods and results in six countries. 2005;1–6. Marie Stopes Internal; Research Brief Series. Available from: https://www.mariestopes.org/media/2124/are-our-voucher-programmes-working-evaluating-methods-and-results-in-six-countries.pdf

[24] Nicole M. Bellows: Vouchers for reproductive health care services in Kenya and Uganda, 2012. Available from: https://www.kfw-entwicklungsbank.de/Download-Center/PDF-Dokumente-Diskussionsbeitr%C3%A4ge/2012_03_Voucher_E.pdf

[25] Borghi J, Ramsey K, Kuwawenaruwa A, Baraka J, Patouillard E, Bellows B, Binyaruka P, Manzi F (2015). Protocol for the evaluation of a free health insurance card scheme for poor pregnant women in Mbeya region in Tanzania: a controlled-before and after study. BMC Health Serv Res.

[26] Wood DJ (1991). Corporate social performance revisited. Acad Manag Rev.

[27] McWilliams A, Siegel D (2001). Corporate social responsibility: a theory of the firm perspective. Acad Manag Rev.

[28] McWilliams A, Siegel DS, Wright PM. Corporate social responsibility: Strategic implications. J Manage Stud. 2006;43(1):1–8. Available from: http://onlinelibrary.wiley.com/doi/10.1111/j.1467-6486.2006.00580.x/full.

[29] Tonin M, Vlassopoulos M. Social incentives matter: Evidence from an online real effort experiment. Available from: https://www.econstor.eu/bitstream/10419/72972/1/736013849.pdf

[30] Lee M, Kohler J (2010). Benchmarking and transparency: incentives for the pharmaceutical industry’s corporate social responsibility. J Bus Ethics.

[31] Georgaraki D. Tax incentives in corporate social responsibility. http://www.cibmp.org/Papers/Paper%20681.pdf.

[32] Drach M, Le Gargasson JB, Mathonnat J, Da Silva A, Kaddar M, Colombini A (2013). EPIVAC international conference on financial sustainability of immunization programs in sub-Saharan Africa, February 16–18, 2012, Ouidah, Benin. Vaccine.

[33] Utzinger J, Wyss K, Moto DD, Tanner M, Singer BH (2004). Community health outreach program of the Chad-Cameroon petroleum development and pipeline project. Clinics in occupational and environmental medicine.

[34] Garvin T, McGee TK, Smoyer-Tomic KE, Aubynn EA (2009). Community–company relations in gold mining in Ghana. J Environ Manag.

[35] Johnson DM, Hokanson DR, Zhang Q, Czupinski KD, Tang J. Feasibility of water purification technology in rural areas of developing countries. J Environ Manag. 2008;88(3):416–27. Available from: http://www.trusselltech.com/uploads/media_items/feasibility-of-water-purification-technology-in-rural-areas-of-developing-countries.original.pdf. 10.1016/j.jenvman.2007.03.002 17459569

[36] Dunfee TW (2006). Do firms with unique competencies for rescuing victims of human catastrophes have special obligations? Corporate responsibility and the AIDS catastrophe in sub-Saharan Africa. Bus Ethics Q.

[37] Corporate responsibility (2003). Shareholders challenge Pepsi over AIDS response in Africa. AIDS policy & law.

[38] Katamba D, TushabomweKazooba C, BabiihaMpisi S, Marvin Nkiko C, Nabatanzi-Muyimba AK, Hensley KJ (2012). Corporate social responsibility management in Uganda: lessons, challenges, and policy implications. Int J Soc Econ.

[39] Alfred A. Living in Kampala Slum: A Socio-economic Analysis in ten informal settlements of Kampala. John Paul II Justice and Peace Centre, Kampala, Uganda. 2011; No.3. Available from: http://jp2jpc.org/downloads/KBNB%202011.pdf.

[40] Amon J, Brown T, Hogle J, MacNeil J, Magnani R, Mills S, Pisani E, Rehle T, Saidel T, Sow CK. Behavioral Surveillance Surveys BSS. Guidelines for repeated behavioral surveys in populations at risk of HIV. Available from: http://www.who.int/hiv/strategic/en/bss_fhi2000.pdf

[41] Garrison LP, Mansley EC, Abbott TA, Bresnahan BW, Hay JW, Smeeding J (2010). Good research practices for measuring drug costs in cost-effectiveness analyses: a societal perspective: the ISPOR drug cost task force report—part II. Value Health.

